# Clinical outcomes with a new microincisional diffractive multifocal IOL

**DOI:** 10.1186/s40662-015-0012-8

**Published:** 2015-01-31

**Authors:** Jorge L Alió, Alfredo Vega-Estrada, Ana B Plaza-Puche

**Affiliations:** Vissum Corporation, Alicante, Spain; Division of Ophthalmology, Universidad Miguel Hernández, Alicante, Spain; Avda de Denia s/n, Edificio Vissum, 03016 Alicante, Spain

## Abstract

**Background:**

To evaluate the refractive outcomes and the optical performance as well as the quality of life in patients implanted with a new diffractive multifocal intraocular lens (IOL).

**Methods:**

Prospective, clinical study including 41 cases of patients who underwent cataract surgery and were divided in two groups: group 1, including 20 eyes implanted with the multifocal IOL SeeLens MF (Hanita Lenses, Israel); group 2, 21 eyes implanted with the Acrysof SA60AT IOL. Visual acuity, defocus curve, intraocular aberrations, contrast sensitivity function and quality of life were assessed during a follow up period of 6 months.

**Results:**

Significant improvement was observed in the uncorrected distance visual acuity (UDVA) and corrected distance visual acuity (CDVA) in both groups (p < 0.02). The multifocal group showed better results in terms of uncorrected near visual acuity (UNVA) and distance-corrected near visual acuity (DCNVA) (p < 0.01). Comparison of both groups showed better visual acuities for the multifocal IOL group in defocus levels from -3.0 D to -1.50 D (p ≤ 0.01). At 6 months, there was a significant reduction of the internal higher order aberrations (p ≤ 0.04). A significant increase in scotopic contrast sensitivity was detected for 6 cycles/° spatial frequency during follow up (p = 0.04), but no significant changes were observed for the rest of spatial frequencies (p ≥ 0.06). Visual Functioning Index (VF-14) questionnaire showed that patients reported high levels of satisfaction when performing daily tasks.

**Conclusions:**

The SeeLens MF IOL is able to successfully restore distance, near and intermediate visions after cataract surgery. It also provides functional intermediate vision with optimal intraocular optical quality.

## Background

The outcomes in terms of quality of life in modern cataract surgery depend in part on the type intraocular lens (IOL) implanted [1-6]. Recent multifocal IOL technology emphasizes refractive and optical quality outcomes aiming to provide distance, intermediate and near-spectacle independence [7-9].

Differences in visual performance achieved with multifocal IOLs depend on the optical principles and IOL designs. Diffractive IOLs are one specific type of multifocal lenses. Many studies [10-17] have reported visual outcomes, optical quality, and quality of life with several models of diffractive IOLs. Refractive results are crucial for the optimal optical performance of this type of IOLs, and control of the refractive astigmatism has a direct impact on this factor. Thus, microincision cataract surgery (MICS) together with IOLs that can be implanted through corneal incisions smaller than 2 mm will show the best outcome in terms of optical performance [10]. There are some studies published in the scientific literature that have reported good outcomes with diffractive IOLs implanted through a sub 2 mm incision [10,13,14]. Recently, a new model of apodized diffractive IOL has been introduced, with an asymmetrical light distribution and the potential to be implanted through a corneal incision smaller than 2.0 mm: the SeeLens MF (Kibbutz Hanita, Israel).

The aim of the current investigation is to evaluate the visual and optical quality outcomes, as well as the postoperative quality of life and clinical outcomes in patients implanted with the new SeeLens MF apodized diffractive multifocal IOL after cataract surgery .

## Methods

### Patients

In this prospective pilot consecutive study, we included 20 eyes of 10 bilateral cataract surgery patients aged 58 to 71 years (mean 67.3 ± 3.8 years) who were implanted with the multifocal IOL SeeLens MF. As a control group, we included 21 eyes of 16 cataract patients aged 46 to 80 years (mean 68.4 ± 9.1 years) with a monofocal IOL implantation. The inclusion criteria of this study were patients with bilateral visually significant cataract, older than 45 years, with corneal astigmatism less than 1.0 D. The exclusion criteria were comorbidities such as, visually significant corneal scars, and known retinal disorders. All patients were adequately informed and signed a consent form. The study adhered to the tenets of the Declaration of Helsinki and was approved by the Vissum Alicante Ethical Board Committee.

### Preoperative examination

Preoperatively, all patients had a full ophthalmologic examination including the evaluation of the refractive status, the distance and near visual acuities, slit lamp examination, tonometry, and funduscopy. Distance and near visual acuity were measured with the ETDRS charts. Other examinations included, corneal topography (CSO, Costruzione Strumenti Oftalmici), and biometry (IOL Master, Zeiss). Finally, in the multifocal IOL group, ocular aberrometry was assessed by means of the KR-1W (Topcon Corp, Tokyo, Japan).

The KR-1W [18] system incorporates three different technologies for the analysis of optical performance of the human eye: wavefront aberrometry using the Hartmann-Shack principle, Placido-disk corneal topography, and standard automatic autorefraction. This system has the advantage of performing the measurement of corneal and global wavefront aberrations on the same axis, therefore, using the same reference for centration in a relatively short time that avoids misalignments from the use of more than one device. Standardized Zernike polynomials were used to reconstruct the wavefront based on the corneal, internal, and whole eye optics. Finally, we also analyzed the impact of the treatment on the quality of the retinal image as analyzed by the Strehl ratio, which provides objective information on optical quality performance at the retinal plane. The KR-1W calculates the Strehl ratio as the relation that exists between the maximum point spread function (PSF) of the real system and the maximum PSF of the perfect system.

IOL power calculation was performed with optical coherence interferometry using the Germany IOL Master (Carl Zeiss Meditec). Target refraction was plano in all cases that were included.

### Surgery

The same surgeon (JLA) performed all surgeries using a standard technique of sutureless microincision (MICS) phacoemulsification. All patients received topical anesthesia before surgery. Adequate dilation was obtained with intracameral mydriasis. The main incision was placed on the axis of the positive corneal meridian. In the group of patients that underwent the procedure with the SeeLens MF, the IOL was implanted in the capsular bag through a corneal incision of 1.8 mm. In the control group, the monofocal IOL were implanted through an incision of 2.0 mm. Postoperative topical therapy included a combination of topical antibiotic and steroid agents (Tobradex® Alcon Cusí Inc, Barcelona).

### The intraocular lenses

The SeeLens MF (Kibbutz Hanita, Israel) is a new C-loop MICS IOL that consist of an aspheric apodized diffractive multifocal IOL. This lens is a single piece IOL with an optic diameter of 6.0 mm, and an overall length of 13.0 mm with a 360° continuous square edge optic. The diffractive steps are located in the 4 mm central zone, suiting pupil sizes in various lighting conditions. The near vision add of this lens is +3.00 D greater than the distance power equivalent to +2.4 D at the spectacle plane. This IOL is made from hydrophilic Acrylic HEMA/EOEMA copolymer and presents as an UV blocker and violet light filter. It has an open C-loop haptic design with a 5° haptic angulation. The overall design of this multifocal IOL allows its implantation through an incision smaller than 2.0 mm, targeting always an incision size of 1.8 mm by using specifically calibrated corneal knives.

The control IOL used in this investigation was the Acrysof SA60AT, which is a 1-piece hydrophobic acrylate–methacrylate copolymer IOL with an anterior asymmetric biconvex optic, and a posterior sharp-edged optic interrupted at the optic–haptic junction and neutral asphericity. It has an optic diameter of 6.0 mm, an overall length of 13.0 mm, and supporting haptics of the same acrylic material as the optic, with 0-degree haptic angulation.

### Postoperative examination

Patients were evaluated during the follow up at 1 day, 1 week, 1 month, 3 months and 6 months after surgery by an experienced optometrist certified in Good Clinical Practice. Distance-corrected near visual acuity (DCNVA) and the intermediate visual acuities were only measured during the postoperative period using the ETDRS test. The postoperative examinations at 1, 3 and 6 months were identical to the preoperative protocol, with additional measurements at 3 and 6 months of the contrast sensitivity function in photopic (85 cd/m^2^) and scotopic (3 cd/m^2^) conditions (CST 1800; Vision Sciences Research Corp, San Ramon, California), and the defocus curve. To generate defocus curves, the visual acuity was measured with the ETDRS charts at 4 m. The defocus curve was obtained in binocular conditions and with best distance correction by adding plus lenses in 0.50 D steps and recording the visual acuity achieved by the patient with each type of blur. Next, the procedure was repeated, but with negative lenses.

At 6 months follow up, an additional examination was performed in the group of patients implanted with the multifocal IOL: the visual quality of life with the Visual Functioning Index (VF-14) questionnaire. The VF-14 asks patients to rate their subjective functional limitations in performing 14 vision-dependent activities of daily living with or without best spectacle corrected vision. Each question had five possible responses graded (0–4).

### Statistical analysis

The statistical analysis was performed with the SPSS software for Windows (version 15.0.1). The average values and standard deviations were calculated for every parameter during the follow up. A non-parametric statistical test, the Wilcoxon Rank Sum test, was applied to assess the significance of differences between preoperative and postoperative data, using in all cases the same level of significance (p < 0.05). The Mann–Whitney test was applied to assess the comparative analysis between groups. For patients who had bilateral surgery, both eyes were considered for the statistical analysis following the recommendations of Karakosta et al. and Armstrong RA [19,20] to elucidate whether data from both eyes could be used as within-subjects factor. An Intraclass Correlation test was carried out to compare data from preoperative biometric parameters. All parameters studied showed a weak correlation (all ICC r0 ≤ 0.34).

### Main outcome measures

Visual, refractive, and defocus curve analyses. The contrast sensitivity function, optical quality assessment, and the quality of life of the patients were also evaluated at the end of the follow up period.

## Results

### Visual and refractive outcomes

Table 1 summarizes the pre- and post-operative visual conditions of the eyes. At 1 month after surgery, a statistically significant improvement was observed in the uncorrected distance visual acuity (UDVA), corrected distance visual acuity (CDVA), uncorrected near visual acuity (UNVA) and corrected near visual acuity (CNVA) (Wilcoxon test, all p < 0.01). No significant changes in these visual parameters were observed in the remaining follow up periods (Wilcoxon test, p ≥ 0.16). DCNVA was 0.22 ± 0.12 (range 0.10 to 0.50), 0.26 ± 0.18 (range 0.0 to 0.80), and 0.15 ± 0.09 (range 0.0 to 0.30) LogMAR at 1, 3 and 6 months postoperatively, respectively. No significant change in this parameter was detected between 1 and 3 months after surgery (Wilcoxon test, p = 0.35), but a significant improvement was found between 3 and 6 months after surgery (Wilcoxon test, p < 0.01). The uncorrected intermediate visual acuity (UIVA) at 63 cm was 0.20 ± 0.13 (range -0.10 to 0.40), 0.24 ± 0.14 (range 0.10 to 0.70), and 0.27 ± 0.15 (range 0.10 to 0.60) LogMAR at 1, 3 and 6 months after surgery, respectively. Distance corrected intermediate visual acuity (DCIVA) at 63 cm was 0.23 ± 0.10 (range 0.10 to 0.40), 0.25 ± 0.14 (range 0.10 to 0.70), and 0.24 ± 0.10 (range 0.10 to 0.40) LogMAR at 1, 3 and 6 months postoperatively, respectively. In addition, the intermediate visual acuity was measured at 100 cm and the outcomes obtained for this distance were: UIVA 0.22 ± 0.12 (range 0.00 to 0.40), 0.25 ± 0.17 (range 0.00 to 0.70), and 0.30 ± 0.15 (range 0.00 to 0.60) LogMAR; DCIVA 0.22 ± 0.10 (range 0.00 to 0.40), 0.23 ± 0.18 (range 0.00 to 0.70), and 0.26 ± 0.12 (range 0.00 to 0.40) LogMAR at 1, 3 and 6 months after surgery, respectively. No significant changes in visual intermediate parameters were observed during the postoperative follow-up (Wilcoxon test, p ≥ 0.09).

**Table 1:** **Visual and refractive outcomes comparison between groups**

**Mean (SD)**	**Preoperative**	**1 Month**	**3 Months**	**6 Months**	**P Value**
**Range**					**Pre-1 Month**
**LogMAR UDVA**	0.73 (0.38)	0.21 (0.15)	0.22 (0.17)	0.22 (0.20)	<0.01
	0.30 to 1.50	0.00 to 0.62	0.00 to 0.70	0.00 to 0.93	
**Sphere (D)**	-0.41 (2.52)	-0.04 (0.59)	0.16 (0.65)	0.10 (0.98)	0.53
	-5.00 to +3.50	-1.00 to +1.50	-0.75 to +2.00	-3.00 to +2.00	
**Cylinder (D)**	-0.78 (0.54)	-0.55 (0.47)	-0.70 (0.55)	-0.81 (0.54)	0.23
	-1.75 to 0.00	-1.25 to 0.00	-1.50 to 0.00	-2.25 to 0.00	
**LogMAR CDVA**	0.33 (0.31)	0.04 (0.05)	0.07 (0.16)	0.04 (0.06)	<0.01
	0.00 to 1.00	0.00 to 0.12	0.00 to 0.70	0.00 to 0.20	
**LogMAR UNVA**	0.69(0.22)	0.24 (0.12)	0.31 (0.22)	0.24 (0.15)	<0.01
	0.40 to 1.00	0.00 to 0.40	0.00 to 0.90	0.00 to 0.60	
**LogMAR CNVA**	0.36 (0.26)	0.13 (0.08)	0.14 (0.14)	0.08 (0.08)	<0.01
	0.10 to 1.0	0.00 to 0.30	0.00 to 0.60	0.00 to 0.30	
**Addition**	2.73 (0.24)	0.75 (0.61)	0.88 (0.81)	0.81 (0.65)	<0.01
	2.50 to +3.00	0.00 to +1.75	0.00 to 2.50	0.00 to +1.50	

Comparative table shows the preoperative and postoperative visual conditions of patients included in this study. The corresponding p-values for the comparison between preoperative and postoperative follow up are shown for each parameter evaluated.

*Abbreviations: SD, standard deviation; D, diopters; UDVA, uncorrected distance visual acuity; CDVA, corrected distance visual acuity; UNVA, uncorrected near visual acuity; CNVA, corrected near visual acuity.

Regarding manifest refraction, no significant changes were found in the sphere and cylinder 1 month after surgery (Wilcoxon test, p ≥ 0.23). A slight trend towards a positive sphere was observed between 1 and 3 months postoperative (from a mean value of -0.04 ± 0.59 D to 0.16 ± 0.65 D (Wilcoxon test, p = 0.03), with no significant modifications afterwards (Wilcoxon test, p = 0.89) (Table 1).

Table 2 summarizes the comparative analysis of visual and refractive outcomes between the multifocal IOL, and the control group preoperatively and 3 months postoperatively. Preoperatively, no statistically significant differences between groups were found in age and IOL power, which indicates that both groups are similar. In contrast, statistical significant differences were detected in postoperative UDVA, CDVA, UNVA, and CDNVA (Mann Whitney tests, p ≤ 0.02) with better UDVA and CDVA for the control group, and with better UNVA and CDNVA for the multifocal IOL group.

**Table 2:** **Comparison of the pre and postoperative variables analyzed in the study**

	**Preoperatively**			**Postoperatively**		
**Mean (SD)**	**Multifocal group**	**Control group**	**P-value**	**Multifocal group**	**Control group**	**P-value**
**Range**						**(Statistical test)**
**Age (years)**	67.30 (3.77)	68.37 (9.11)	0.65	---	---	---
	58 to 71	46 to 80				
**LogMAR UDVA**	0.73 (0.38)	1.40 (0.74)	<0.01	0.22 (0.17)	0.13 (0.14)	0.02
	0.30 to 1.50	0.10 to 2.00		0.00 to 0.70	0.00 to 0.50	
**Sphere (D)**	-0.41 (2.52)	-0.61 (4.70)	0.92	+0.16 (0.65)	+0.03 (0.48)	0.4
	-5.00 to +3.50	-7.25 to +12.00		-0.75 to +2.00	-1.00 to +1.25	
**Cylinder (D)**	-0.78 (0.54)	-0.80 (0.46)	0.93	-0.70 (0.55)	-0.59 (0.56)	0.37
	-1.75 to 0.00	-1.75 to 0.00		-1.50 to 0.00	-2.25 to 0.00	
**LogMAR CDVA**	0.33 (0.31)	0.24 (0.22)	0.51	0.07 (0.16)	0.02 (0.04)	0.02
	0.00 to 1.00	0.00 to 0.82		0.00 to 0.70	0.00 to 0.20	
**LogRAD UNVA**	0.69 (0.22)	0.71 (0.34)	0.64	0.31 (0.22)	0.57 (0.16)	<0.01
	0.40 to 1.00	0.10 to 1.10		0.00 to 0.90	0.30 to 0.90	
**LogRAD CDNVA**	---	---	---	0.26 (0.18)	0.60 (0.12)	<0.01
				0.00 to 0.80	0.40 to 0.89	
**LogRAD CNVA**	0.36 (0.27)	0.26 (0.19)	0.15	0.14 (0.14)	0.15 (0.07)	0.77
	0.10 to 1.20	0.10 to 0.70		0.00 to 0.60	0.10 to 0.30	
**IOL Power (D)**	20.80 (1.87)	21.74 (4.98)	0.5	---	---	---
	18.0 to 23.50	14.0 to 40				

Comparative table shows the preoperative and postoperative conditions at 3 months after cataract surgery. The corresponding p-values for the comparison between groups are shown for each parameter evaluated.

*Abbreviations: SD, standard deviation; D, diopters; UDVA, uncorrected distance visual acuity; CDVA, corrected distance visual acuity; UCNVA, uncorrected near visual acuity; CNVA, corrected near visual acuity.

### Defocus curve

**Figure 1:**
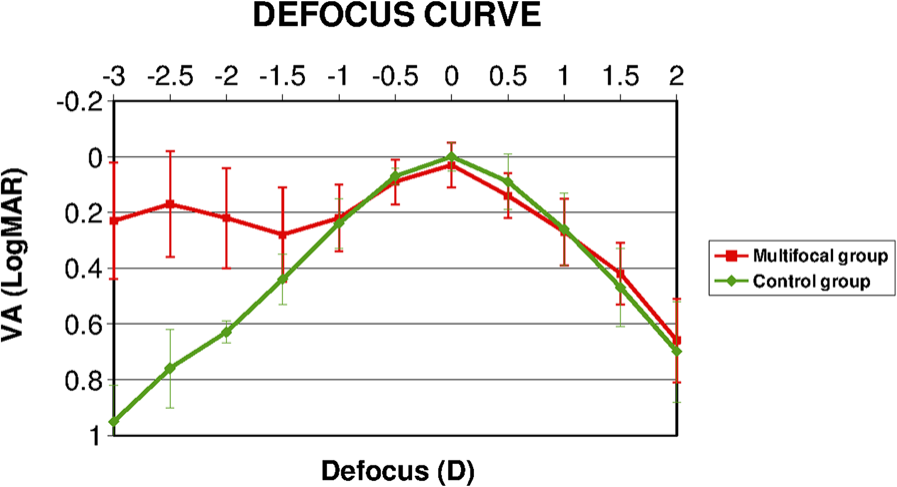
**Defocus curve comparison between groups.** Comparison of mean defocus curve between the patients implanted with the SeeLens MF IOL and the monofocal IOL.

Figure 1 shows the mean defocus curve of the patients analyzed in the current study. It was found that this multifocal IOL provided a bimodal profile showing two peaks of maximum vision, one at distance (around 0 defocus level), and one at near (around -2.5 D defocus level). Between these two peaks, defocus of approximately -1.5 D was deemed to provide acceptable intermediate vision (better than 0.3 LogMAR). When the multifocal IOL group was compared with the monofocal IOL control group, statistically significant differences were observed in defocus levels from -3.0 D to -1.50 D with better visual acuities for the multifocal IOL group (Mann Whitney tests, p ≤ 0.01).

### Contrast sensitivity function

**Figure 2:**
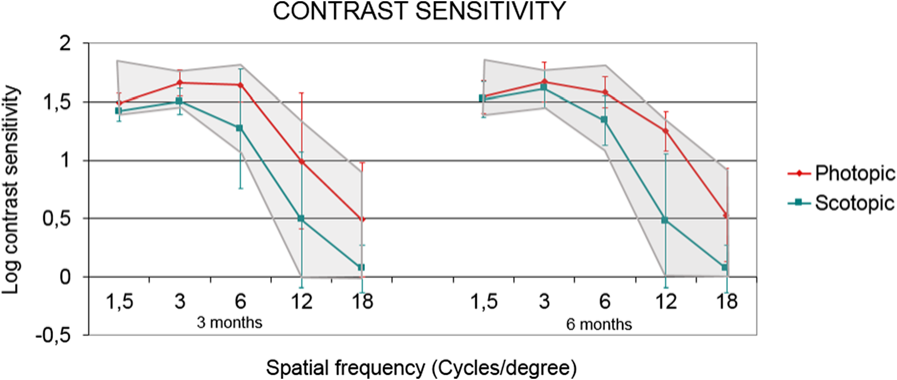
**Contrast sensitivity function in the multifocal IOL group.** Mean postoperative contrast sensitivity function (CSF) in patients implanted with the multifocal IOL in logarithmic scale under photopic and scotopic conditions at 3 and 6 months after surgery. Normal values for the same age group are shown in light gray.

Figure 2 shows the mean postoperative contrast sensitivity function (CSF) in logarithmic scale under photopic and scotopic conditions at 3 and 6 months after surgery for the group of patients implanted with the multifocal IOL. A significant increase in scotopic contrast sensitivity was detected for 6 cycles/° spatial frequency during follow-up (Wilcoxon test, p = 0.04), but no significant changes were observed for the rest of spatial frequencies (Wilcoxon test, p ≥ 0.06). Figure 2 also shows, in light gray, the normal levels of CSF for patients of the same age. It can be observed that the results of the CSF after implantation of the SeeLens MF are within physiological level for patients of the same age group.

**Figure 3:**
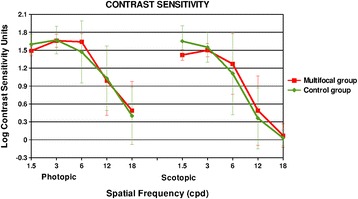
**Contrast sensitivity function comparison between groups.** It shows comparison of the postoperative contrast sensitivity function in both groups of patients under photopic and scotopic conditions.

Figure 3 shows the comparison of the CSF between both groups of patients in photopic and scotopic conditions 3 months after the surgery. It was observed that patients implanted with the monofocal IOL for the spatial frequencies corresponding to 1.5 cycles/° in scotopic conditions had slightly better behaviors, but these differences were not statistically significant (p > 0.05).

### Optical quality assessment

**Figure 4:**
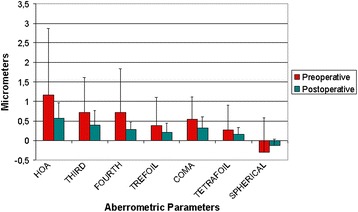
**Internal aberrations in the multifocal IOL group.** It shows evolution of the internal aberrations throughout the follow up period. There is a significant reduction for the root mean square (RMS) of the internal high order aberrations six months after implantation of the SeeLens MF IOL.

Figure 4 shows the internal aberrometric outcomes. At 6 months after surgery, there was a significant reduction for the root mean square (RMS) of the internal high order aberrations and in the coma aberration (Wilcoxon test, p ≤ 0.04). Also, a significant reduction for the RMS for the third and forth order aberrations was detected (Wilcoxon test, both p = 0.03). However, no significant changes were observed in the internal trefoil, tetrafoil and spherical aberrations (Wilcoxon test, both p ≥ 0.41).

Regarding the optical quality analysis, a significant increase of the ocular Strehl ratio was observed from 0.11 ± 0.06 preoperative to 0.19 ± 0.11 at 6 months after surgery (Wilcoxon test, p = 0.02).

### Quality of life outcomes

Table 3 summarizes the achieved mean quality of life outcomes in patients implanted with the multifocal IOL. These data were obtained with the VF-14 questionnaire at 6 months after surgery. Patients had more difficulty driving at night, and reading small print, such as medicine bottle labels, a telephone book, or food labels.

**Table 3:** **Results of the VF-14 quality of life questionnaire in the multifocal IOL group**

**Items**	**Punctuation**
1. Reading small print, such as medicine bottle labels, a telephone book, or food labels	1.00 ± 0.93
2. Reading a newspaper or a book	0.50 ± 0.53
3. Reading a large-print book or large-print newspaper or numbers on a telephone	0.13 ± 0.35
4. Recognizing people when they are close to you	0.33 ± 0.71
5. Seeing steps, stairs or curbs	0.11 ± 0.33
6. Reading traffic signs, street signs or store signs	0.11 ± 0.33
7. Doing fine handwork like sewing, knitting, crocheting, carpentry	0.75 ± 0.89
8. Writing checks or filling out forms	0.63 ± 0.74
9. Playing games such as bingo, dominos, card games, or mahjong	0.00 ± 0.00
10. Taking part in sports like bowling, handball, tennis, golf	0.00 ± 0.00
11. Cooking	0.00 ± 0.00
12. Watching television	0.22 ± 0.44
13. Driving during the day	0.20 ± 0.45
14. Driving at night	1.20 ± 0.45

Mean values of the VF-14 QOL questionnaire items at 6 months postoperatively. Grading scale: 0, no difficulty; 1, a little difficulty; 2, moderate difficulty; 3, quite difficult; 4, impossible to perform.

### Surgical and postoperative complications

No postoperative complications were observed, specifically, no significant posterior capsule opacification causing visual decrease of 1 or more lines associated with visual symptoms and leading to neodymium-doped yttrium aluminum garnet (Nd:YAG) laser capsulotomy. No significant IOL decentration was detected at the slit lamp examination either.

## Discussion

Restoring the functional near vision in presbyopic patients who undergo cataract surgery has become an important outcome goal of refractive surgeons. The loss of near functional vision negatively impacts the quality of life of presbyopic patients [21]. During the last two decades, several designs of multifocal IOL technologies have been developed in order to provide adequate spectacle independence for patients after cataract surgery. However, some limitations related to the design of the optic and the distribution of the light within the different foci have led to the presence of undesirable symptoms in some cases such as, the presence of photic phenomena, and the reduction of the contrast sensitivity function [11,22,23].

The present investigation evaluated the clinical outcomes, as well as the optical performance, and the quality of life in patients that underwent implantation of a new type of diffractive IOL for the correction of presbyopia, the SeeLens MF (Kibbutz Hanita Lenses, Kibbutz, Israel).

Previously published studies that have evaluated the results of multifocal IOLs have reported an improvement in the visual acuity at different distances after cataract surgery and refractive lens exchange [10,12,14,15,24]. In the present investigation, we found similar results to those previously reported by other authors. We observed a significant improvement in the different ranges of vision that were evaluated using the SeeLens MF IOL [6,10,12,13]. Although most patients achieved near and distance functional visual acuity with different models of diffractive IOLs, a main limitation is the poor intermediate vision that this technology provides [22]. Therefore, several multifocal IOL manufacturers have developed optics that includes aspheric profiles with low addition in order to improve functional intermediate vision [12,15,23]. The SeeLens MF IOL incorporates both an aspheric profile, and an addition of 3 diopters that allows the patients a wide range of reading performance at intermediate distances as the current investigation shows. In addition, the results derived from the defocus curve show that the design of this IOL provides two excellent peaks of maximum vision for the focus corresponding to the distance and the near visions with a slight slope between these two peaks, suggesting that the patients can achieve an adequate and functional intermediate visual acuity. One of the reasons that could explain this behavior may be related to the fact that the new design of this IOL is based on an aspheric refractive-diffractive apodized profile.

Measurement of the intraocular aberrations demonstrated a significant reduction in the intraocular higher order aberrations, and in the asymmetric aberrations (coma and coma-like aberrations). These results are similar to those previously reported by our research group when analyzing the intraocular optical quality with different diffractive surfaces [12,16], and by other studies that have evaluated the intraocular optical performance of multifocal IOLs [24,25]. Although it was not statistically significant, we found a change towards a more positive spherical aberration. This might be related to the aspheric profile on the SeeLens MF, which introduces a negative aspheric factor within the optic of its design.

This study also evaluated the quality outcomes of the patients by measuring the quality of the image in the retinal plane. We found that after the implantation of the SeeLens MF IOL, the patients achieved better levels of Strehl ratio to those shown in the preoperative period. In addition, the mean postoperative Strehl ratio was better than those observed in a normal population of the same age and was comparable to values obtained in young, healthy patients [26]. Moreover, Strehl ratios in patients that underwent implantation of the SeeLens MF were better than those previously reported by our research group with other types of diffractive IOLs [16]. In addition, a recent study in which also analyzed the outcomes of patients implanted with the SeeLens MF, the authors reported statistically better results in terms of visual quality when comparing with another diffractive multifocal IOL [27]. We have to take into account the fact that the intraocular optical quality in the present investigation was evaluated with a Hartmann-Shack aberrometer, thus, the outcomes presented in the current investigation should be taken with caution as this kind of wavefront technology has shown to be limited when evaluating the diffractive surface [28].

Regarding the contrast sensitivity function, it was observed that six months after the surgery, the SeeLens MF provides results in photopic condition and on the higher spatial frequencies that are within the physiological levels for the normal population of the same age group [29]. Nevertheless, analysis of the results in scotopic condition and in the remaining spatial frequencies showed that there was a reduction of the CSF after surgery. Similar findings were reported in other studies that have evaluated the outcomes related to the CSF after implantation of diffractive IOL models, which have also found a reduction of the CSF in the spatial frequencies that were analyzed [15]. The decrease in contrast sensitivity in patients implanted with multifocal IOL is due to the dispersed distribution of light energy within the surface of the optic [30], which is usually more pronounced in low light conditions as our results showed. Another reason that explains the reduction of CSF with this type of multifocal IOL is the relationship that exists between the optical quality and the near visual performance of the IOL. Thus, the better the near vision provided by the IOL, the greater the limitation that will exist in terms of visual quality [16]. Even though there is a reduction of the CSF after the surgical procedure, we found that there is a trend to obtain better contrast perception of the image between 3 and 6 months follow up. This improvement relates to the quality of the image perceived, and should be attributed as a possible effect of the neuroadaptation process that was observed in those patients implanted with multifocal IOLs [31].

Finally, the present investigation also assesses the quality of life by analyzing the answer of the patients to the VF-14 questionnaire [32]. Even though patients found more difficulty in driving at night, and reading small print, such as medicine bottles or food labels, most of the answers to the questionnaire showed the high levels of satisfaction that the patients had when asked about the tasks that they often perform in their daily lives. These findings are consistent with those found with the other variables analyzed in the present investigation, confirming the ability of this IOL in improving the quality of life of the patients. Additionally, van der Linden et al. also showed in their investigation a high rate of satisfaction in patients implanted with the SeeLens MF [27]. In that study, as many as 96% of the subjects that were evaluated reported to be satisfied with the result of the procedure.

In the current study, a control group, which underwent a monofocal IOL implantation, was also included with the aim of comparing the outcomes with those observed in the SeeLens MF IOL group. Both groups presented similar preoperative characteristics and therefore could be compared without significant statistical bias. As expected, an improvement in both, corrected and uncorrected vision were observed after the surgery in the two groups of patients. In terms of near vision, the SeeLens MF provided a better near visual outcome than the monofocal IOL, confirming the efficacy of this IOL in restoring near visual function. Regarding the defocus curve, it was demonstrated that the patients implanted with the multifocal IOL were able to achieve two peaks of maximum vision, one at distance and one at near with a good intermediate vision. On the other hand, those patients implanted with the monofocal IOL only showed one peak of maximum vision. These findings demonstrate that the SeeLens MF IOL provides a range of functional vision for different distances in comparison with those that can be achieved with a monofocal IOL.

Another aspect that is worth discussing is the one related to MICS that is defined by those phacoemulsification procedures that can be performed through an incision smaller than 1.5 mm [33]. This type of surgery offers several advantages such as a stable anterior chamber during surgery, a reduced amount of ultrasound power, less surgical induced astigmatism, among others. Nevertheless and despite the aforementioned advantages, one of the limitations of this procedure is that there are few multifocal IOLs available in the market that can be implanted through an incision of less than 2 mm. On the other hand, stability and centration of multifocal diffractive IOL optics is critical for the adequate optical performance of this technology. Thus, a tilt or decentration can decrease the visual quality and produce optical side effects, causing subjective symptoms and patient dissatisfaction [34]. Hence, an IOL design is crucial to the optimum optical function, and to prevent problems that can lead to vision complaints. The SeeLens MF incorporates in its design, an open C-loop haptic that provides stability of the IOL inside the capsular bag. Previous reports in the scientific literature have demonstrated that IOLs that showed the best performances in terms of stability inside the capsular bag are those with C-loop haptic designs [35]. To the best of our knowledge, the SeeLens MF is currently the only multifocal diffractive IOL with available scientific data published in the literature, which can provide both the capability to be implanted through an incision smaller than 2 mm and a C-loop design of the haptics.

## Conclusions

In conclusion, the MICS SeeLens MF IOL can restore distance and near visions in presbyopic patients undergoing cataract surgery. This new IOL also provides functional intermediate vision with an adequate intraocular optical quality performance. In addition, the results obtained from the quality of life questionnaire confirm the high levels of satisfaction in the patients implanted with the SeeLens MF IOL. Finally, by providing both, the capability of being implanted through a 1.8 mm incision, and the stability due to the open C-loop haptic design, the SeeLens MF IOL offers an excellent alternative for modern cataract surgical techniques. Further long-term studies with a larger sample of patients should be performed in order to confirm the outcomes observed in this investigation with the new SeeLens MF IOL.
